# Breast-conserving surgery with or without radiotherapy in women with ductal carcinoma in situ: a meta-analysis of randomized trials

**DOI:** 10.1186/1748-717X-2-28

**Published:** 2007-08-02

**Authors:** Gustavo A Viani, Eduardo J Stefano, Sérgio L Afonso, Lígia I De Fendi, Francisco V Soares, Paola G Leon, Flavio S Guimarães

**Affiliations:** 1 Department of Radiation Oncology, Faculty of Medicine of Marília (FAMEMA), Marília, São Paulo, Brazil; 2 Department of Radiation Oncology, Instituto Nacional de Enfermedades Neoplásicas, Lima, Perú; 3 Department of Radiation Oncology, Hospital A.C.Camargo, São Paulo, Brazil

## Abstract

**Background:**

To investigate whether Radiation therapy (RT) should follow breast conserving surgery in women with ductal carcinoma in situ from breast cancer (DCIS) with objective of decreased mortality, invasive or non invasive recurrence, distant metastases and contralateral breast cancer rates. We have done a meta-analysis of these results to give a more balanced view of the total evidence and to increase statistical precision.

**Methods:**

A meta-analysis of randomized controlled trials (RCT) was performed comparing RT treatment for DCIS of breast cancer to observation. The MEDLINE, EMBASE, CANCERLIT, Cochrane Library databases, Trial registers, bibliographic databases, and recent issues of relevant journals were searched. Relevant reports were reviewed by two reviewers independently and the references from these reports were searched for additional trials, using guidelines set by QUOROM statement criteria.

**Results:**

The reviewers identified four large RCTs, yielding 3665 patients. Pooled results from this four randomized trials of adjuvant radiotherapy showed a significant reduction of invasive and DCIS ipsilateral breast cancer with odds ratio (OR) of 0.40 (95% CI 0.33 – 0.60, p < 0.00001) and 0.40 (95% CI 0.31 – 0.53, p < 0.00001), respectively. There was not difference in distant metastases (OR = 1.04, 95% CI 0.57–1.91, p = 0.38) and death rates (OR = 1.08, 95%CI 0.65 – 1.78, p = 0.45) between the two arms. There was more contralateral breast cancer after adjuvant RT (66/1711 = 3.85%) versus observation (49/1954 = 2.5%). The likelihood of contralateral breast cancer was 1.53-fold higher (95% CI 1.05 – 2.24, p = 0.03) in radiotherapy arms.

**Conclusion:**

The conclusion from our meta-analysis is that the addition of radiation therapy to lumpectomy results in an approximately 60% reduction in breast cancer recurrence, no benefit for survival or distant metastases compared to excision alone. Patients with high-grade DCIS lesions and positive margins benefited most from the addition of radiation therapy. It is not yet clear which patients can be successfully treated with lumpectomy alone; until further prospective studies answer this question, radiation should be recommended after lumpectomy for all patients without contraindications.

## Background

Ductal carcinoma in situ (DCIS, intraductal carcinoma, noninvasive duct carcinoma) of the breast represents a heterogeneous group of proliferative lesions with diverse malignant potential, and a range of controversial treatment options. It is the most rapidly growing subgroup of breast cancer, with over 55,000 new cases diagnosed in the United States in 2003[1]. DCIS has been traditionally classified according to architectural pattern (ie, comedo, cribriform, micropapillary, papillary and solid types). However, this classification was developed at a time when mastectomy was the recommended treatment, and histological classification was largely an academic exercise. With the increasing use of breast conservation therapy (BCT), there is a need to identify lesions that are more likely to recur locally, and thus, might be better treated with more aggressive therapy. Local control is the predominant issue for breast conserving approaches because in the absence of invasive disease, distant or regional recurrences are not an issue. Grade and histological subtype have been the most widely studied predictive factors for local failure after treatment with BCT. High grade lesions, particularly those of the comedo subtype, are more likely to recur locally than are low grade lesions [2-8]. As our knowledge of DCIS has evolved, the treatment decision-making process has become more complex and controversial. The lack of a single appropriate treatment option for DCIS is reflected in national patterns of care [9,10]. The variability in therapy is illustrated by findings from the Surveillance, Epidemiology, and End Results (SEER) database of the National Cancer Institute [10]. Among 25,206 patients treated for DCIS among 1992 to 1999, mastectomy rates decreased from 43 to 28 percent, and utilization of radiation therapy (RT) following lumpectomy remained near 50 percent. Although no randomized trials comparing BCT directly with mastectomy have been completed, the available data suggest that both provide similar cause-specific survival in patients with DCIS. Furthermore, in those who elect BCT, randomized trials have shown a reduction in the risk of a local in-breast recurrence with RT, although no survival benefit compared to excision alone and there does not appear to be a selective benefit for RT in preventing invasive recurrences. Thus, the need for RT as a component of BCT in women with DCIS is controversial. Clinical trials have shown that local excision and RT in patients with negative margins can produce excellent rates of local control [11-16]. However, RT as a component of BCT may represent overly aggressive therapy, since the majority of cases of DCIS do not recur or progress to invasive cancer when treated with excision alone [3,17-19]. In this way, the aim of our meta-analysis is to summarizes the results of randomized trials performed, to evaluate the real impact of adjuvant radiotherapy in patients with DCIS to reduce in situ recurrence, invasive breast recurrence, distant metastasis, death rates and to identify one subgroup of patients who no need of adjuvant RT.

## Methods

### Types of studies

This meta-analysis properly included randomized controlled clinical trials. Any trial including only patients with DCIS or any trial including patients with DCIS which stratify by absence/presence of DCIS and where patients with DCIS but not invasive cancer can be separated out were included. The participants of studies included women diagnosed with DCIS for the first time, not recurrent or metastatic disease with no prior history of malignant disease (other than in situ carcinoma of the cervix, or BCC or SCC of skin) without invasive breast cancer and no age limit. The intervention criteria for to be include in this review was any trial in which radiotherapy (of any kind) was the primary adjuvant treatment comparison to breast conserving surgery (lumpectomy, quadrantectomy, segmental mastectomy) without radiotherapy. The efficacy of the outcomes evaluated in our study was if adjuvant radiotherapy reduced new DCIS (ipsilateral/contralateral breast), invasive breast cancer ipsilateral, contralateral breast, distant metastasis and death rates.

### Search strategy for identification of studies

Medline and manual searches were done (completed independently and in duplicate) to identify all published (manuscripts and abstracts) randomized controlled trials (RCTs) that compared adjuvant radiotherapy for DCIS breast cancer to observation. The Medline search was done on PubMed between 1966 and 2006 with no language restrictions, using the search terms "ductal carcinoma in situ," breast cancer" and "observation," adjuvant radiotherapy" or "post operative radiotherapy," and "breast conserving surgery" (lumpectomy, quadrantectomy and segment mastectomy). The second search was done through CancerLit, and the Cochrane Library to identify randomized trials published between January 1998 and July 2006, using MeSH headings (ductal carcinoma in situ, adjuvant radiotherapy, observation, breast cancer/sc {Secondary}, ex-lode Clinical Trials, clinical trial {publication type}) and text words (ductal carcinoma in situ, adjuvant treatment:, radiotherapy, trial, and study) without language restrictions. All the searched abstracts were screened for relevance. Manual searches were done by reviewing articles and abstracts cited in the reference lists of identified RCTs, by reviewing the first author's article, abstract file, from reference lists of retrieved papers, textbooks and review articles. Also, abstracts published in the Proceedings of the Annual Meetings of the American Society of Clinical Oncology (through 2005) were systematically searched for evidence relevant to this meta-analysis. The selection of studies for inclusion was carried out independently by two of the authors (V-GA and E-JS). Study suitable was assessed using QUOROM criteria [20]. Each study was evaluated for quality using the scale of 1 to 5 proposed by Jadad [21]. If reviewers disagreed on the quality scores, discrepancies were identified and a consensus was reached. Trial data abstraction was also done independently and in duplicate, but abstractors were not blinded to the trials' authors or institution. Any discrepancies in data abstraction were examined further and resolved by consensus.

### Analysis of the review

The data analyses were made with Review Manager Version 4.2 provided by The Cochrane Collaboration. All analyses were carried out on an intention-to-treat basis; that is, all patients randomly assigned to a treatment group were included in the analyses according to the assigned treatment, irrespective of whether they received the treatment or were excluded from analysis by the investigators. For categorical variables, weighted risk ratios and their 95% confidence interval were calculated using RevMan 4.2 software according to the Peto method [22]. Results were tested for heterogeneity at significance level of P < 0.05 according to the methods outlined by Der Simonian and Laird [23]. A fixed effects model was used if there was no evidence of heterogeneity between studies, if there was evidence of heterogeneity random effects model was used for meta-analysis. The odds ratio and 95% confidence interval were calculated for each trial and presented in a Forrest plot. Sensitivity analyses was performed by excluding the trials which Jadad-scale was only 1 score. Publication bias is a common concern in meta-analysis that is related to the tendency of journals to favor the publication of large and positive studies. We chose a commonly used method for detecting publication bias, which is a graphical plot of estimates of the ORs from the individual studies versus the inverse of their variances, which is commonly referred to as a "funnel plot." An asymmetry in the funnel would be expected if there was publication bias with smaller studies tending to show larger ORs, because small studies with no significant statistical results would be less likely to be reported. Differences in mortality, ipsilateral invasive recurrence, ipsilateral DCIS recurrence, distant metastasis, brain metastases or contralateral breast cancer recurrence were collected. Mortality was defined as death from any cause, ipsilateral recurrence was defined as recurrence of invasive or DCIS breast cancer at same breast treated by RT and contralateral breast cancer recurrence was defined as recurrence in the breast no treated by RT; Distant metastases was defined as of the first distant tumor recurrence, ignoring locoregional recurrences and second breast or non breast cancers.

## Results

### Description of trials

**Figure 1:**
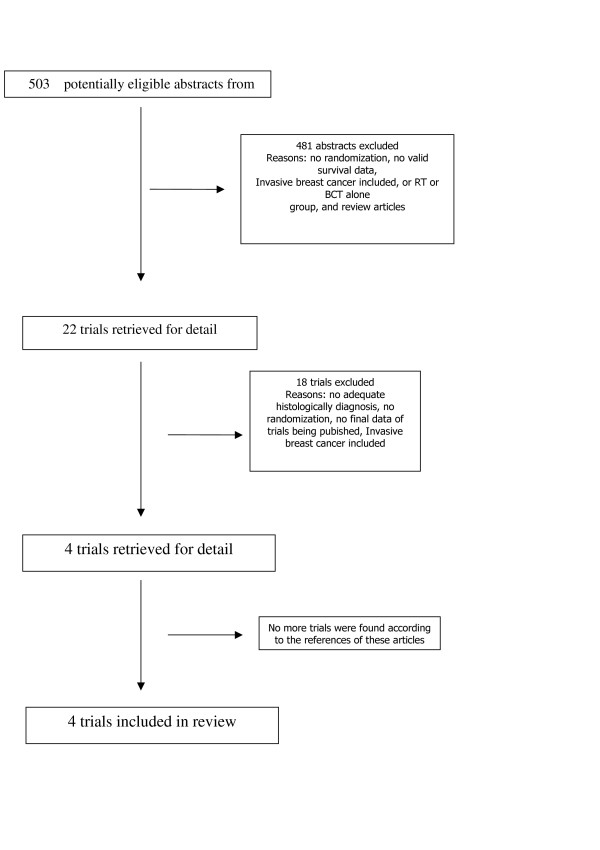
The flowchart. RT: radiotherapy; RCTs: randomized controlled trials, BCT: Breast conserving therapy.

The two trial assessors agreed on the selection of four randomized controlled trials fulfilled the eligibility criteria. The Quorum flow diagram illustrates the main reasons for trial exclusion (Figure 1)[11,13,15,24]. Combining these trials yielded data on 3665 patients, 1711 and 1954 patients were submitted to RT and BCT alone, respectively. These four prospective randomized trials have been performed to define the Role of radiation therapy in the management of DCIS with breast conservation (Table 1). These are NSABPB-17 [11], EORTC10853 [13], the UK Coordinating Committee on Cancer Research (UKCCCR) trial [15] and SWE DCIS [24]. Two arms of UKCCCR were not included in this review, due to use of tamoxifen alone or in association with radiotherapy. The characteristics of RCTs how: duration of the study, total dose and fractionation of RT, median follow up, number of patients, percentage of central pathological review and the patterns of recurrence are resumed in table 1 and 2.

**Table 1:** Trials of radiotherapy following local excision for DCIS

Trial	NSABP-17	EORTC10853	UKCCCR	SWE-DCIS
**Characteristic**				
**Date**	1985–1990	1986–1996	1990–1998	1987–1999
**Patients Randomised**	818	1010	1030	1046
**Median follow-up**	12 YEARS	4 YEARS	4.3 YEARS	5.2 YEARS
**Symptomatic**	19%	28%	NA	12.9%
**Central path review**	76%	85%	79%	20%
**Dose**	50 Gy/25 Fraction	50 Gy/25 Fractions	50 Gy/25 Fractions	50 – 54 Gy/25–27 fractions
**Quality by Jadad**	4	4	4	4
**Population**	Pre- and post-menopausal Pts. All pts had tumour free margins after BCT. Women with localized ductal carcinoma in situ detected by physical examination or mammography were eligible, both ductal carcinoma in situ and lobular carcinoma in situ were also eligible. RT 411 pts BCT403 pts	DCIS<5 cm 1 002/1 010 pts analysed Extent of free margins was not specified, evidence of invasive carcinoma or Paget's disease of the nipple, were ineligible for the study. RT 502 pts BCT 500 pts	Screening detected tumor, complete excision of the carcinoma, free margins. typical ductal hiperplasia excluded. Excluded patients with lobular carcinoma in situ or atypical ductal hyperplasia in the absence of ductal carcinoma in situ, those in whom pathological margins of disease were uncertain, and people with Paget's disease of the nipple. RT 267 BCT 544	Pathology margins clear, DCIS grade I and II B/2 mm were classified as atypical ductal hyperplasia Exclusion criteria were Paget's disease of the nipple, invasive carcinoma or intracystic carcinoma in situ, ongoing pregnancy or a history of previous or concurrent malignancy RT 534 BCT 533

RT = radiotherapy; BCT = breast conserving treatment; DCIS = ductal carcinoma in situ

**Table 2:** Comparison of breast cancer events in prospective randomized trials of DCIS treatment

	**EORTC 10853 [13]**		**NSABP B17 [11]**		**SWE DCIS [24]**		**UK/ANZ DCIS [15]**	
	4 y follow up		4 follow up		5.2 follow up		4.3 y follow-up	

	L	L+RT	CL	L+RT	L	L+RT	L	L +RT

Type of cancer	(n = 503)	(n = 507)	(n = 403)	(n = 410)	(n = 533)	(n = 534)	(n = 544)	(n = 267)
**Ipsilateral breast cancer**								
Total	83	53	98	49	117	44	115	22
Invasive	40	24	66	29	69	23	39	10
No invasive	44	29	32	20	48	21	76	12
**Contralateral Breast cancer**								
Total	8	21	8	10	22	26	11	9
Invasive	5	16	5	8	15	23	6	9
No invasive	3	5	3	2	7	3	5	0

L = Lumpectomy, RT = radiotherapy

### NSABPB-17

In NSABP B-17, 818 women with DCIS were randomly assigned to excision alone or followed by RT (50 Gy to the whole breast). The main endpoint was local recurrence, invasive or intraductal. Histologically negative surgical margins were required in both groups, although inking of margins was not required, and a margin could be interpreted as clear if as few as three collagen fibers separated the DCIS from an inked surface. This trial was initiated in 1985 at a time when knowledge of DCIS was limited. Neither prospective mammographic-pathologic correlation nor lesion sizing was performed, and resected tissue was only sampled histologically. After 12 years of follow-up, the cumulative incidence of invasive and noninvasive ipsilateral breast tumors combined was 31.7% in the lumpectomy-alone arm and 15.7% in the lumpectomy-plus-radiation arm (P.001). The 12-year overall survival was 86% for patients in the lumpectomy group and 87% for patients in the lumpectomy and radiation therapy group (P.08).

### EORTC10853

EORTC trial 10853, which was identical in design to NSABP B-17, randomly assigned 1010 women with completely resected, mammographically detected DCIS ≤5 cm to postoperative RT (50 Gy in five weeks) or no further therapy [13]. After a median follow-up of 4.25 years, local recurrence was documented in 17% of patients in the lumpectomy-alone arm and 11% in the lumpectomy-plus-radiation arm. Patients with free margins had little difference in local recurrence rates with the addition of radiation therapy (12% versus 14%). In patients with close or involved margins, the addition of radiation to lumpectomy reduced the local recurrence rate from 32% to 16% [13]. In the latest report with average 10-year follow-up, the group receiving RT had significantly fewer invasive (8 versus 13 percent) and noninvasive (5 versus 14 percent) recurrences as compared to surgery alone.

### UKCCCR

In the United Kingdom, Australia, New Zealand DCIS Trial (UK/ANZ Trial), 1701 women who underwent excision of DCIS with clear margins were randomly assigned to RT (yes or no), and/or to tamoxifen versus placebo, using a two by two factorial design [15]. This yielded four subgroups: excision alone, excision plus RT, excision plus tamoxifen, and excision plus RT plus tamoxifen. The primary outcome was the incidence of subsequent ipsilateral invasive breast cancer. The complex study design used in this trial makes interpretation of the data somewhat difficult. With a median follow-up of 53 months, those who underwent RT had a significantly lower risk of ipsilateral invasive (HR 0.45) and intraductal recurrence (HR 0.36), similar in magnitude to the results from the NSABP and EORTC. There were new breast events in 7% of patients in the radiotherapy group and 16% of those in the no-radiotherapy group (P.001).

### SWE DCIS

This trial studied the effect of postoperative radiotherapy (RT) after breast sector resection for ductal carcinoma in situ (DCIS). The study protocol stipulated radical surgery but microscopically clear margins were not mandatory. SWE DCIS randomized 1046 operated women to postoperative RT or control between 1987 and 1999. The primary endpoint was ipsilateral local recurrence. Secondary end points were contralateral breast cancer, distant metastasis and death. After a median follow-up of 5.2 years (range 0.1–13.8) there were 44 recurrences in the RT group corresponding to a cumulative incidence of 0.07 (95% confidence interval (CI) 0.050 – 10). In the control group there were 117 recurrences giving a cumulative incidence of 0.22 (95% CI 0.18 – 0.26) giving an overall hazard ratio of 0.33 (95% CI 0.24 – 0.47, p < 0.0001). Twenty two percent of the patients had microscopically unknown or involved margins, no evidence for different effects of RT on the relative risk of invasive or in situ recurrence. Secondary end points did not differ. All these studies compared excision with recommended negative margins versus the same surgery and addition of whole breast radiotherapy at 50 Gy in 25 fractions without a boost [24].

### Overall mortality

**Figure 2:**
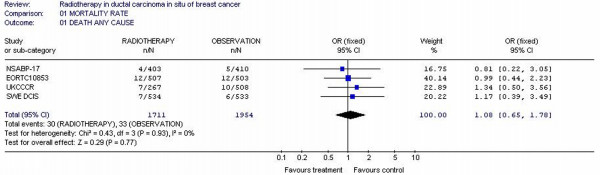
Meta-analysis examining adjuvant radiotherapy in DCIS for patients with breast cancer: ipsilateral DCIS breast cancer recurrence.

All the studies reported overall survival as one of the outcomes. Altogether, the analyses included 4 trials with 3665 patients. The overall mortality rates were not decreased for RT arm (30/1711 = 1.75%) compared to observation arms (33/1954 = 1.68%). The individual odds ratios ranged from 0.22 to 3.56 with a pooled odds ratio for all of the trials of 1.08 with a 95% confidence interval of 0.65 to 1.78. The test for heterogeneity was not statistically significant with p value 0.93, which indicates that the pooling of the data was valid. The overall odds ratio suggests that there is no difference between RT arms and observation arms in terms of overall mortality rate with p value 0.77. RT arms was not superior to observation in decreased mortality rate in none study. No observation arms reaching any statistical significance in decreased mortality rate. (figure 2)

### Ipsilateral DCIS Recurrence rate

**Figure 3:**
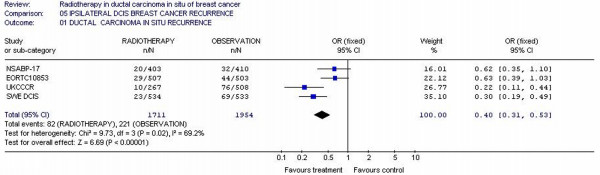
Meta-analysis examining adjuvant radiotherapy in DCIS for patients with breast cancer: ipsilateral invasive breast cancer recurrence.

Four studies [11,13,15,24] reported this outcome representing a total of 3665 patients. The ipsilateral DCIS recurrence rates were 4.79% (82/1711) and 11.3% (221/1954) for RT arms and observation arms, respectively. The individual odds ratios varied from 0.30 to 0.62. The test for heterogeneity was statistically significant (p = 0.02). Repeated analyses of the above end points using random effect did not alter the results and conclusion. The overall odds ratio was 0.40 (95% CI 0.31 – 0.53) with p value <0.00001, which suggests that there was difference for ipsilateral DCIS recurrence between the RT and observation arms for adjuvant therapy. (figure 3)

### Invasive ipsilateral breast cancer

**Figure 4:**
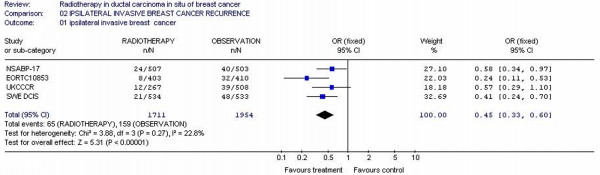
Meta-analysis examining adjuvant radiotherapy in DCIS for patients with breast cancer: ipsilateral invasive breast cancer recurrence.

Four studies [11,13,15,24] had reported invasive ipsilateral breast cancer data and 3665 patients were included in the analysis. There were more invasive ipsilateral breast cancer for observation (159/1954 = 8.1%) compared to RT arms (65/1711 = 3.8%). The likelihood of invasive ipsilateral cancer was 0.40-fold smaller (95% CI 0.33 – 0.60) in RT arms patients. Test for heterogeneity was not significant with p value of 0.27 (figure 4)

### Distant metastasis rates

**Figure 5:**
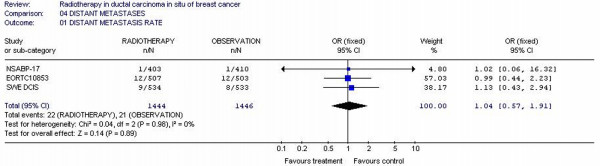
Meta-analysis examining adjuvant radiotherapy in DCIS for patients with breast cancer: distant metastases.

Only three studies [11,13,24] reported the metastases rates. Two thousand, eight hundred and ninety patients were randomized in these three studies. The metastases rates for all randomized patients were not different; 1.52% (22/1444) for radiotherapy patients and 1.45% (21/1446) for observation patients (p = 0.89). Tests for heterogeneity in the analysis were not significant (p = 0.98). (Figure 5)

### Contralateral breast recurrence

**Figure 6:**
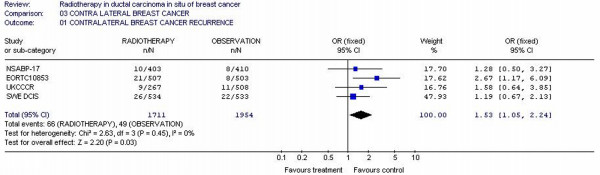
Meta-analysis examining adjuvant radiotherapy in DCIS for patients with breast cancer: contralateral breast cancer recurrence.

Four studies [11,13,15,24] with 3665 patients reported contralateral breast recurrence incidence rate. The likelihood of contralateral recurrence was 1.53-fold higher (95% CI 1.05 – 2.24) in RT arms patients, with p value of 0.03, which suggests that there was difference for ipsilateral contralateral breast cancer recurrence between the RT and observation arms for adjuvant therapy. Test for heterogeneity was not significant with p value of 0.45, which indicates that the pooling of the data was valid (Figure 6).

### Quality of studies

The RCT quality scores was of 4 point (5-point scale), the median score was 4 of 5 with none study scoring 0, 1, 2, or 3. There was complete agreement in scoring by the two assessors. The quality scores were high and the same (n = 4) for the all trials. This fact occurred because of the importance placed on blinding in the scoring system, and the inherent difficulty in blinding a treatment such as radiation.(table 1)

### Evaluation of Publication Bias

**Figure 7:**
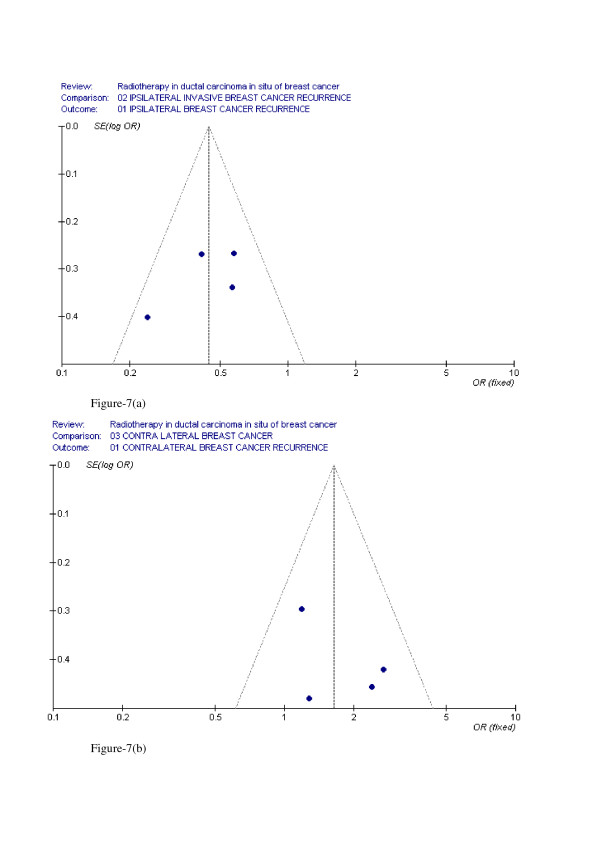
(a) funnel plot for ipsilateral invasive breast cancer recurrence (b) funnel plot for contralateral breast cancer recurrence.

The funnel plot of the log ORs versus the inverse of their variances of the individual studies is displayed in Figure 7a–7b. The plot formed a very distinct funnel shape with the log ORs evenly distributed around the meta-analysis OR regardless of the study variance. Therefore, there was no indication of an asymmetry in the study findings by the variance or size of the studies and, thus, little evidence for publication bias.

## Discussion

DCIS represents a broad biologic spectrum of disease, and a wide range of treatment approaches have been proposed. The lack of clear and universally accepted treatment selection criteria has resulted in a diverse array of confusing clinical recommendations. The need for RT as a component of BCT in women with DCIS is controversial. Clinical trials have shown that local excision and RT in patients with negative margins can produce excellent rates of local control [11-16]. Our meta-analysis of four trials that evaluated adjuvant radiotherapy in 3665 patients with CDIS submitted to BCT showed that adjuvant RT leads to a significant reduction (60%) in the risk of a local (invasive and DCIS) in-breast recurrence. However, RT as a component of BCT may represent overly aggressive therapy, since the majority of cases of DCIS do not recur or progress to invasive cancer when treated with excision alone [3,17-19]. The results of NSABP B-17 have been used to justify radiation for all women; however, pathologic factors affecting local control were largely unrecognized when this and other prospective studies were designed and initiated. Furthermore, deficiencies in pathologic evaluation likely resulted in underdiagnosis of invasive disease and margin involvement. As an example, because neither NSABP B-17 nor EORTC 10853 provided mammographic correlation with either the preoperative imaging and/or with specimen radiography, the completeness of excision is uncertain. In NSABP B-17, microscopic tumor size was not determined, sampling of the surgical specimen could not reliably exclude invasive disease or involved margins, and, as noted previously, management of the resection margins was suboptimal. Reflecting these inadequacies, local recurrence rates were 16 percent at 12 years in the irradiated group [12]. Similar long-term results were noted in a collaborative study of women undergoing BCT with RT for DCIS in ten institutions in the United States and Europe (15-year actuarial breast recurrence rate 19 percent [25]). Such data suggest significant limits on the ability of RT to control residual DCIS in the breast, and underscores the importance of complete pathologic specimen evaluation. These deficiencies limit the ability to extrapolate the trial results to patients who undergo optimal pathologic evaluation. Prospective but nonrandomized studies with a larger focus on pathology, including mammographic correlation, have achieved at least comparable results without RT in identifiable subsets of patients with DCIS [15,26-29]. By weighing factors of prognostic importance (ie, grade, size, age, and margin width), a sizable subset of patients with DCIS (32 percent of those treated by BCT in one study [27]) can be identified who have a 99 percent twelve-year local recurrence-free survival with excision alone. However, the routine use of these factors, which are included in the USC/Van Nuys Prognostic Index (USC/VNPI) requires a higher standard of pathologic practice than is reflected in the published randomized trials. The original USC/VNPI assigned scores of 1, 2, or 3 for histological type, width of the surgical margin, and lesion size [26]. A prospectively collected but nonrandomized study suggested that local recurrence rates for lesions with USC/VNPI scores 3 to 4 were acceptably low with excision alone (local recurrence-free survival rates exceeded 99 percent at eight years of follow-up), while those with intermediate scores (5 to 7) required the addition of RT to achieve optimal local control. In contrast, RT provided a significant degree of benefit for the subset of patients whose DCIS was characterized by high grade, large size, and narrow margins (USC/VNPI 8 to 9). The recurrence rate was 100 percent at three years without RT, compared to 60 percent at eight years with RT. Although the relative benefit was large, neither outcome was deemed clinically acceptable, and it was recommended that such patients might be better served by mastectomy. Thus, the identification of subgroups of patients with DCIS for whom RT offers little absolute benefit in local recurrence-free survival is of clinical relevance [30]. RT is time consuming, and accompanied by significant side effects in a small percentage of patients (eg, cardiac, pulmonary, second malignancies). Radiation fibrosis can alter the texture of the breast and skin, making mammographic follow-up more difficult [31]. Ongoing trials by both the European and the United States cooperative groups are addressing the issue of benefit from RT. One of them, performed by Radiation Therapy Oncology Group study (RTOG 9804) aims to randomly assign 1790 patients either to radiation therapy or observation, with the option of tamoxifen in either group. The patients must have lesions that are 2.5 cm or less in diameter, low- or intermediate-grade nuclei, and inked margins that are at least 3 mm in diameter. The primary outcomes will be the difference in local recurrence and distant disease-free survival rates. The recently closed European Cooperative Oncology Group (ECOG E-5194) trial was similar. It accrued approximately 1000 patients with DCIS lesions that were 2.5 cm or less and low- or intermediate-grade nuclei or 1 cm or less and high-grade nuclei (both groups had to have negative margins that were at least 3 mm). Patients were treated by lumpectomy alone, and the primary outcome will be local recurrence rates at 5 and 10 years. The results of these studies should provide information on the efficacy of lumpectomy alone with good-risk DCIS and may allow the development of criteria to identify subgroups of patients who do not require adjuvant radiation therapy. Moreover, our data evidenced that contralateral tumor rates are slightly increased by radiotherapy (Figure 3). For the four trials combined there were 49 contralateral tumors in the surgery alone compared to 66 in the patients who also received radiotherapy (P = 0.03). Studies investigating the induction of breast cancer after radiotherapy for Hodgkin's disease have shown intervals of at least 8 years between the primary treatment and presentation of subsequent breast cancer. A large increase was seen in the first report of the EORTC trial [13], though this may represent a superior finding, given the low standard doses used and short follow up. It has been suggested that the method of delivery of RT in the EORTC study may explain this finding [32] but has been somewhat reduced with longer follow-up [33,34]. The first analysis of the NSABP B-17 did not report a difference in the occurrence of contralateral breast cancer, although in their 8-year update a higher, but non-significant rate of contralateral breast cancer in the treatment group was found when only those contralateral breast cancers occurring as first events (13 in the no further treatment group *vs *19 in the radiotherapy group) were included. However, when all contralateral breast cancers were included in the analysis, this difference disappeared (18 *vs *20)[11]. Studies investigating breast-conserving therapy for invasive breast cancer have not reported an increased rate of contralateral breast cancer. Although our results may have occurred by chance these studies will monitor this finding, if the increase is due to radiotherapy, it will reduce the overall value of this form of therapy for DCIS patients. Finally, none of these trials was sufficiently powered to detect differences in overall survival. Similar rate of distant metastases and death were observed in the two groups. The risk of eventually dying from breast cancer when DCIS is treated with breast-conserving treatment is still no clear. In our study 63 patients died from breast cancer. Although this does not seem a high rate, the follow-up time was relatively short. A few of these deaths would probably have occurred even if the patients had initially had a mastectomy. However, given the number of invasive local recurrences, a significant number will probably die from metastatic disease. Longer follow-up is needed to investigate whether the beneficial effect of radiotherapy on local control will improve survival rates.

## Conclusion

DCIS represents a heterogeneous group of intraductal clonal proliferations of varying malignant potential. Although the rate of local (in breast) recurrence is substantially higher with breast conservation, survival following a local recurrence is excellent. Our data demonstrate a reduction in the risk of a local in-breast recurrence (invasive and CDIS) with RT, although no survival benefit compared to excision alone. Thus, the identification of subgroups of patients with DCIS for whom RT offers little absolute benefit in local recurrence-free survival is of clinical relevance. However, no data based in RCTs are currently available to identify a subgroup of women with the kind of DCIS who did not need to be treated with radiation therapy. In this way, news trials lead for cooperative groups would have to investigate benefits from RT.

## Competing interests

The author(s) declare that they have no competing interests.

## Authors' contributions

VG carried out the search, acquisition and interpretation of the data in studies. He also drafted the manuscript. VG performed the statistical analysis and drafted the manuscript. SE participated in the design of the study, carried out the search for articles and gave final approval of the version to be published. S LA and S FV participated in the design of the study, L PG gave final approval of the version to be published, D LI gave final approval of the version to be published, G FS participated in the design of the study and gave final approval of the version to be published. All authors read and approved the final manuscript.
